# Prediction of Recurrent Venous Thromboembolism by Clot Lysis Time: A Prospective Cohort Study

**DOI:** 10.1371/journal.pone.0051447

**Published:** 2012-12-11

**Authors:** Ludwig Traby, Marietta Kollars, Lisbeth Eischer, Sabine Eichinger, Paul A. Kyrle

**Affiliations:** 1 Department of Medicine I, Medical University of Vienna, Vienna, Austria; 2 Karl Landsteiner-Institute of Clinical Thrombosis Research, Vienna, Austria; Leiden University Medical Center, The Netherlands

## Abstract

Venous thromboembolism (VTE) is a chronic disease, which tends to recur. Whether an abnormal fibrinolytic system is associated with an increased risk of VTE is unclear. We assessed the relationship between fibrinolytic capacity (reflected by clot lysis time [CLT]) and risk of recurrent VTE. We followed 704 patients (378 women; mean age 48 yrs) with a first unprovoked VTE for an average of 46 months after anticoagulation withdrawal. Patients with natural coagulation inhibitor deficiency, lupus anticoagulant, cancer, homozygosity for factor V Leiden or prothrombin mutation, or requirement for indefinite anticoagulation were excluded. Study endpoint was symptomatic recurrent VTE. For measurement of CLT, a tissue factor-induced clot was lysed by adding tissue-type plasminogen activator. Time between clot formation and lysis was determined by measuring the turbidity.135 (19%) patients had recurrent VTE. For each increase in CLT of 10 minutes, the crude relative risk (RR) of recurrence was 1.13 (95% CI 1.02–1.25; p = 0.02) and was 1.08 (95% CI 0.98–1.20; p = 0.13) after adjustment for age and sex. For women only, the adjusted RR was 1.14 (95% CI, 0.91–1.42, p = 0.22) for each increase in CLT of 10 minutes. CLT values in the 4^th^ quartile of the female patient population, as compared to values in the 1^st^ quartile, conferred a risk of recurrence of 3.28 (95% CI, 1.07–10.05; p = 0.04). No association between CLT and recurrence risk was found in men. Hypofibrinolysis as assessed by CLT confers a moderate increase in the risk of recurrent VTE. A weak association between CLT and risk of recurrence was found in women only.

## Introduction

Approximately a third of patients with first unprovoked venous thromboembolism (VTE) have recurrence of VTE within 5 years after completion of anticoagulant therapy [1]. The case-fatality rate of recurrent VTE ranges between 4 and 12% [1]. Current guidelines recommend extended anticoagulation in patients with unprovoked proximal deep vein thrombosis (DVT) or pulmonary embolism (PE) provided they have a low bleeding risk and coagulation monitoring can be accomplished at regular intervals [2]. This recommendation, however, implies that many patients who do not experience recurrence are unnecessarily exposed to a bleeding risk. Various diagnostic strategies have been applied to separate high-risk patients, who might benefit from indefinite anticoagulation, from low-risk patients, who will possibly not. Determination of single laboratory risk factors of recurrence has turned out to be futile. A better stratification of patients with regard to their recurrence risk can be achieved by measuring coagulation activation markers such as D-Dimer or in vitro thrombin generation [1].

Only few studies have focussed on a possible association between hypofibrinolysis and recurrent VTE. In a small retrospective study, Korninger found a higher incidence of recurrent DVT or superficial phlebitis among patients with a euglobulin lysis time (ELT) longer than 60 minutes [3]. In the Duration of Anticoagulation (DURAC) Study, increased levels of plasminogen activator inhibitor type 1 and tissue–type plasminogen activator (tPA) antigen correlated with the development of recurrent VTE within the next 3 to 6 years [4]. Crowther et al. found no difference in the levels or activity of type 1 plasminogen activator, tPA or ELT between patients who did, or did not suffer recurrent thrombosis [5]. In our study in patients with a first unprovoked VTE, the probability of recurrence was 2-fold higher among patients with thrombin-activatable fibrinolysis inhibitor (TAFI) levels exceeding the 75^th^ percentile [6]. In the Leiden Thrombophilia Study, no association was found between clot lysis time (CLT) or TAFI antigen levels and recurrent VTE [7].

The purpose of this analysis was to investigate if hypofibrinolysis is associated with an enhanced risk of recurrent VTE. Rather than distinct components of the fibrinolytic system, we measured CLT, which we regard a global marker of fibrinolytic capacity [8].

## Methods

### Patients and Study Design

This analysis was performed within the frame of the Austrian Study on Recurrent Venous Thromboembolism (AUREC), a large prospective ongoing multi-centre cohort study. Between January 2000 and September 2008 consecutive patients with a first unprovoked DVT of the leg and/or PE who had been treated with anticoagulants for 3 to 18 months were included. The present analysis considers follow-up data until June 2010. Exclusion criteria were: age younger than 18 years; cancer at time of enrolment; or requirement for long-term antithrombotic treatment for other reasons than VTE. Patients entered the study at the time of discontinuation of anticoagulation. At study entry, a detailed medical history was obtained, and a physical examination was performed. Three weeks after withdrawal of anticoagulation, they were screened for biochemical and genetic risk factors of VTE. Patients were excluded when they had deficiency of antithrombin, protein C, or protein S; presence of the lupus anticoagulant; or when they were homozygous or double heterozygous for factor V Leiden and/or the G20210A prothrombin mutation. At the same time, aliquots of plasma were stored at −80°C.

Patients were seen at six-month intervals for the first year and once a year thereafter. They were given detailed written information on symptoms of VTE and were asked to report immediately if such symptoms occurred. A medical history was obtained at each visit. Female patients were strongly discouraged from intake of estrogen-containing oral contraceptives or hormone replacement therapy. Patients received thromboprophylaxis with a low-molecular-weight heparin during high-risk situations such as surgery, trauma, prolonged immobilization or long-haul air travel. The ethics committee of the Medical University of Vienna approved the study and all patients gave written informed consent prior to inclusion in the study.

### Diagnosis of VTE

As previously described, the diagnosis of VTE was established by a positive finding on venography, colour duplex sonography, ventilation-perfusion lung scanning or spiral computed tomography (CT) [9].

### Study End Points

The end point of the study was recurrent symptomatic DVT confirmed by venography or colour duplex sonography, or recurrent symptomatic PE confirmed by multi-slice CT, ventilation-perfusion lung scanning or autopsy. Recurrent DVT was diagnosed if the patient had an abnormal compression ultrasound or an intraluminal filling defect on venography in case of documented recanalisation of the initial thrombus, a thrombus in another deep vein in the extremity involved in the previous event, a thrombus in the opposite extremity, or a thrombus in the same venous system with a proximal extension of the thrombus (if the upper limit of the original thrombus had been visible) or the presence of a constant filling defect surrounded by contrast medium (if the original thrombus had not been visible).

### Laboratory Analysis

Venous blood from fasting patients was collected into EDTA tubes or in 1∶10 volume of 0.11 mM trisodium citrate and immediately centrifuged for 20 min at 2000×*g*. CLT was determined in plasma obtained three weeks after completion of anticoagulation and at a time when the prothrombin time was normal. Frozen plasma was thawed in a water bath at a temperature of 37°C for 10 minutes. The plasma was then transferred onto a 96-well microtiter plate (50 µl per well). 50 µl of a previously prepared reaction mixture consisting of 9325 µl Hepes buffer (137 mM NaCl +3,5 mM KCl +3 mM CaCl2+25 mM Hepes +0,1% BSA, pH 7,4), 20 µl Innovin (final dilution 1∶1000, Dade Behring, Marburg, Germany), 340 µl CaCl (of 1 M solution), 115 µl tPA (10 µg/ml solution, Hyphen Bio Med, Neuville-sur-Oise, France) and 200 µl phospholipide [solution of 28% phosphatidylserine, 42% phosphatidylcholin and 30% sphingomyelin (total lipid concentration 0,5 mmol/L), Rossix, Mölndal, Sweden] was added to every well using a multichannel pipette. The final concentration per well was 17 mM CaCl2, 56 ng/ml tPA, 10 µmol/l phospholipid. The plate was thoroughly mixed and then incubated at 37°C in an absorbance microplate reader (ELx808, Biotek, USA). The optical density at 405 nm was monitored every 30 seconds, resulting in a clot-lysis turbidity profile. Screening for factor V Leiden and the G20210A prothrombin mutation, and measurement of antithrombin, protein C and protein S were carried out by standard methods. The diagnosis of the lupus anticoagulant was based on criteria of the International Society on Thrombosis and Haemostasis [10].

### Statistical Analysis

Categorical data were compared among groups using contingency-table analyses (the chi-square test). Continuous data (presented as means ± SD) were compared by means of Mann-Whitney U tests. Survival-time methods were used to analyse the time to recurrence among patients with a subsequent episode of VTE (uncensored observation) or the duration of follow-up among patients without recurrence (censored observations) [11]. Data on patients who left the study or who were lost to follow-up were censored at the time of withdrawal. The probability of recurrence was estimated according to the method of Kaplan and Meier [12]. To test for homogeneity between strata, we applied the log-rank test. Univariate and multivariate Cox proportional-hazards model were used to analyse the association between CLT and the risk of recurrent VTE. All P values were two-tailed, and were considered as indicating statistical significance if lower than 0.05. SPSS software, version 15.0, was used.

## Results

### Patient Characteristics

The study population consisted of 704 patients with a first unprovoked VTE (Table 1). The average age was 47 years, and 54% were women. After the previous episode of VTE, the patients had received anticoagulants for an average of almost 8 months. 172 patients (24%) were carriers of factor V Leiden, and 37 patients (5%) had the G20210A prothrombin mutation. The mean CLT was 73.6±15.1 minutes. After the discontinuation of treatment with anticoagulants, the patients were followed for an average of 46 months. A total of 121 patients left the study because they required antithrombotic treatment for causes other than venous thrombosis (65 patients), or because they were given a diagnosis of cancer (16), because they became pregnant (29) or for other reasons (11). 36 patients were lost to follow-up. One patient died of an ischemic stroke, one of cardiac failure, and one patient committed suicide. Data on these 3 patients were censored at the time of death.

**Table 1:** Characteristics of the 704 patients.

Sex		
	Women n (%)	378 (54%)
	Men n (%)	326 (46%)
Age (yrs)		47±15
Location of first VTE; n (%)		
	DVT	318 (45%)
	DVT+PE	195 (28%)
	PE	191 (27%)
Factor V Leiden; n (%)		172 (24%)
G20210A prothrombin mutation; n (%)		37 (5%)
Mean CLT (min)		73.6±15.1
Duration of anticoagulation (mo)		7.6±2.5
Observation time (mo)		46±30

### Recurrence of VTE

135 patients of the 704 patients (19.2%) had a recurrent VTE. 69 patient (51%) had DVT only and 66 patients (49%) had PE with or without DVT. PE was fatal in 3 patients. Recurrence was unprovoked in 117 patients (87%). The probability of recurrence was 5.9% (95% CI, 4.1–7.7), 10.4% (95% CI, 8.0–12.8) and 21.5% (95% CI, 17.8–25.2) after 12, 24 and 60 months, respectively. Recurrent VTE occurred more often in men (29.8%) than in women (10.1%).

### CLT and the Risk of Recurrent VTE

Patients with recurrent VTE had longer CLTs than those without a recurrence (76.2±16.9 vs. 73.0±14.6 minutes; p = 0.1*).* When CLT was analyzed as a continuous variable in a Cox proportional-hazards model, the relative risk (RR) of recurrence was 1.13 (95% CI, 1.02 to 1.25; P<0.02) for each increase in the CLT of 10 minutes. After adjustment for age, the RR was 1.12 (95% CI 1.00–1.24; p = 0.047) and was 1.08 (95% CI 0.98–1.20; p = 0.13) after additional adjustment for sex.

We next evaluated whether the relationship between CLT and recurrent VTE was linear or whether there was a threshold level of CLT for an increase in the recurrence risk. We calculated the RR associated with each of several different ranges of CLT levels (Table 2). The RR gradually increased from the 2^nd^ to the 4^th^ quartile of CLT values. The risk of recurrence was more than 1.6 times greater among patients with CLT equal or longer than the 75^th^ percentile of the values in the study population as among patients with levels in the reference range (below the 25^th^ percentile). After adjustment for age and sex, the RR was 1.4-fold among patients in the 4^th^ quartile, but was no longer statistically significantly increased.

**Table 2:** Relative risk of recurrent VTE according to quartiles of CLT.

CLT (min)	Patients (n)	Recurrences (n)	RR (95% CI)	RR (95% CI)*
≤63.5	173	24	1.0 (ref.)	1.0 (ref.)
>63.5–71.5	174	35	1.49 (0.89–2.51)	1.33 (0.79–2.24)
>71.5–81	177	37	1.56 (0.94–2.62)	1.42 (0.85–2.38)
>81	180	39	1.67 (1.00–2.78)	1.39 (0.83–2.31)

* adjusted for age and sex.

### CLT and the Risk of Recurrent VTE – Effect of Sex

Our findings suggested an interaction between CLT, sex and the risk of recurrent VTE. Men had significantly longer CLTs than women (76.1±16.2 minutes vs. 71.5±13.8 minutes; p<0.001) and were older than women (52±13 years vs. 50±17 years; p<0.001). No difference between men and women was found with regard to duration of anticoagulation, observation time and frequency of factor V Leiden or G20210A prothrombin mutation carriership (data not shown). Women aged 50 years or less had significantly shorter CLTs as compared to women older than 50 years (68.1+13.3 minutes vs. 77.3+12.6 minutes, p<0.001).

When CLT was analyzed as a continuous variable in a Cox proportional-hazards model for women only, the RR of recurrence was 1.2 (95% CI, 0.98–1.46, p = 0.07) and was 1.14 (95% CI, 0.92–1.42, p = 0.22) after adjustment for age. No such association was found in men [RR 1.04 (95% CI, 0.92–1.18); p = 0.52].

Women with CLTs equal to or longer than 61.5 minutes had a more than 2-fold higher risk of recurrent VTE than women with CLT values in the first quartile (Table 3). CLT values in the 4^th^ quartile, as compared to values in the 1^st^ quartile, conferred a 2.7-fold risk of recurrence after adjustment for age.

**Table 3:** Relative risk of recurrent VTE according to quartiles of CLT in women.

CLT (min)	Patients(n)	Recurrences(n)	RR(95% CI)	RR(95% CI)*
≤61.5	92	4	1.0 (ref.)	1.0 (ref.)
>61.5–69.7	97	11	2.80 (0.89–8.80)	2.64 (0.84–8.33)
>69.7–79.5	94	10	2.35 (0.74–7.49)	2.07 (0.64–6.69)
>79.5	95	13	3.28 (1.07–10.05)	2.69 (0.85–8.55)

* adjusted for age.

## Discussion

In this prospective cohort study of more than 700 patients with a first unprovoked VTE the risk of recurrent VTE was as high as 21% 5 years after withdrawal of anticoagulants. The principal finding was that hypofibrinolysis was associated with a moderate although statistically not significant increase in the risk of recurrent VTE. We used CLT as a global marker of fibrinolytic capacity and found that patients with CLT values in the 4^th^ quartile of the patient population had a more than 1.6-fold greater recurrence risk compared to the reference group, i.e. patients with CLT values in the 1^st^ quartile. The increased risk of recurrence was, however, seen only in women: women with CLT values in the 4^th^ quartile had a 3-fold greater risk of recurrence compared to women with short CLT values.

Data on the association between hypofibrinolysis and recurrent VTE are scarce and conflicting. Inconsistent study outcomes may well be explained by differences between patient populations, study designs and laboratory test systems. Our findings are in line with our previous analysis performed in the same patient cohort [6]. We found a 2-fold greater recurrence risk in patients with TAFI (which is an important determinant of the CLT assay [8]) levels exceeding the 75^th^ percentile compared with lower values. The risk was highest among patients with both high TAFI and high plasma levels of factors VIII, IX or XI. Our findings are also in agreement with data reported by Korninger et al. already several years ago. In this retrospective study of only 121 patients, the recurrence rate was 4.8%/year in patients with a post-occlusion ELT shorter than 60 minutes and was 10.3%/year in those with a longer ELT [3]. In the DURAC Study, the proportion of patients, who had t-PA antigen levels above the normal range, was higher among those who experienced recurrence after a first or second VTE compared to those who did not (59% vs. 34%, p<0.001). When the t-PA antigen levels were adjusted for age, no difference between patients with or without recurrence was seen [4]. Of note, a large proportion of patients in this study had VTE secondary to a temporary risk factor or more than 2 episodes of VTE. 2 studies, one from Canada and one from the Netherlands [5], [7], found no association between various fibrinolytic variables and risk of recurrent VTE. In the former study, however, fibrinolytic testing was performed in only a small number of patients after anticoagulant therapy had been discontinued, and both patients with provoked and unprovoked VTE were studied. In the latter study, patients older than 70 years of age and patients with PE were excluded. Many patients in this study experienced the index VTE event in the presence of a temporary risk factor and thus have to be regarded as low risk patients unlikely to benefit from indefinite anticoagulation. With the exception of our own study [3], none of the aforementioned reports took a possible difference in fibrinolytic variable levels between men and women into consideration.

Our study has strengths and limitations. With more than 700 consecutive VTE patients (of whom 135 experienced objectively documented recurrence) and a follow-up of almost 4 years in average, our study is one of the largest today. Our study population is homogenous as we only included patients with a first unprovoked VTE. In contrast to patients with secondary VTE, this is the patient population with a high risk of recurrence that might benefit from extended anticoagulation. The patients are seen in our department at regular intervals and the number lost to follow-up is low. Our findings, however, cannot be extrapolated to patients with a provoked VTE or to those with a strong thrombophilic defect, because these patients were excluded and treated on a routine basis for a longer period of time. The CLT assay used in this study employs exogenous tPA to effect lysis. Consequently, the test provides no indication of endogenous tPA activity. Our study is a hypothesis-generating cohort study, which precludes predefinition of certain cutoff values.

What is the clinical relevance of our findings? The optimal duration of secondary thromboprophylaxis entails balancing the risk of recurrent VTE against the risk of bleeding related to anticoagulation. Identification of distinct patients with a recurrence risk that is high enough to justify institution of long-term anticoagulation would allow individualized patient management. Determination of single components of the coagulation system, i.e. laboratory thrombophilia screening, has failed in this respect [1]. A more promising approach consists in measuring global coagulation markers, the level of which might reflect multicausal thrombophilia. Indeed, patients can be stratified into high and low risk categories with respect to their recurrence risk by measuring D-Dimer or thrombin generation shortly after cessation of anticoagulation [1]. D-Dimer levels have been incorporated in 2 prediction models of recurrent VTE [13], [14], both of which, however, require validation before routine use. In this respect, our findings regarding the potential usefulness of a global indicator of fibrinolytic capacity (such as the CLT) to stratify thrombosis patients according to their recurrence risk are inconclusive. Although we found an association between CLT and risk of recurrent VTE, this relationship is not strong enough to serve as a solid basis on which clinical decisions regarding duration of anticoagulation can be made. It remains, however, to be seen if by combining CLT with other global coagulation markers, for instance D-Dimer or in vitro thrombin generation, or by incorporating CLT in prediction models, discrimination between high and low risk patients in terms of their recurrence risk could be improved.
